# Machine learning based on radiomics for discriminating sellar region langerhans cell histiocytosis from germ cell tumors

**DOI:** 10.3389/fped.2026.1775150

**Published:** 2026-04-24

**Authors:** Hongting Jiang, Zanyong Tong, Yu Luo, Zhenxian Li, Lanxue Shi, Lusheng Li, Yuting Zhang

**Affiliations:** 1 Department of Radiology, Children’s Hospital of Chongqing Medical University, National Clinical Research Center for Children and Adolescents’ Health and Diseases, Ministry of Education Key Laboratory of Child Development and Disorders, International Science and Technology Cooperation Base of Child Development and Critical Disorders, Chongqing Key Laboratory of Child Neurodevelopment and Cognitive Disorders, Chongqing, China; 2 Department of Neurosurgery, Children’s Hospital of Chongqing Medical University, National Clinical Research Center for Children and Adolescents’ Health and Diseases, Ministry of Education Key Laboratory of Child Development and Disorders, International Science and Technology Cooperation Base of Child Development and Critical Disorders, Chongqing Key Laboratory of Child Neurodevelopment and Cognitive Disorders, Chongqing, China

**Keywords:** germ cell tumors (GCTs), langerhans cell histiocytosis (LCH), machine learning, magnetic resonance imaging (MRI), radiomics, sellar region tumors

## Abstract

**Background:**

Differentiating sellar region germ cell tumors (GCTs) from Langerhans cell histiocytosis (LCH) is challenging due to highly similar MRI features, especially in tumor marker-negative patients. In this study, we aimed to develop and validate a radiomics model to distinguish tumor marker-negative sellar GCTs from LCH.

**Methods:**

This retrospective study enrolled a total of 93 patients diagnosed pathologically or by therapeutic diagnosis at our single institution between April 2012 and April 2024, including 40 cases of LCH and 53 cases of GCTs. Radiomics features extracted from multiparametric MRI, including T1-weighted imaging (T1WI) and T2-weighted imaging (T2WI). We manually segmented the regions of interests (ROIs) of tumors. Feature selection was subsequently performed using LASSO regression with five-fold cross-validation. We have chosen three machine learning classifiers-Support Vector Machine (SVM), Random Forest (RF), and Logistic Regression (LR) to construct models based on the 7 features which were retained. Additionally, by integrating clinically significant features and imaging semantic features, classification models based on radiomics features, imaging semantic features, and clinical features were developed separately. There are 7 models in total. Furthermore, combined prediction models were constructed based on different fusion feature sets, respectively. The performance of the diagnostic model was evaluated using the receiver operating characteristic (ROC) curve. The mean area under the curve (AUC), sensitivity, specificity, accuracy, and F1-score were calculated for both the development set and the test set. Differences in AUC between models were assessed using DeLong's test, and the resulting *P*-values were adjusted using the Bonferroni false discovery rate (FDR) correction method. Code available upon request.

**Results:**

The best diagnostic performance was achieved by the combined model of radiomics with clinical features and imaging semantic features using the RF classifier, with an AUC value of 0.81. A statistically significant difference (*p* < 0.05) was confirmed by the DeLong test, indicating robust diagnostic capability.

**Conclusion:**

Radiomics-based machine learning is a promising, non-invasive approach to distinguish tumor marker-negative sellar GCTs from LCH, which has good predictive performance and may help with treatment decision-making.

## 1 Introduction

Intracranial germ cell tumors (ICGCTs) are relatively rare malignant neoplasms, accounting for approximately 3% of all childhood central nervous system (1). They predominantly affect children and adolescents, with an overall male-to-female ratio of about 2:1. These tumors commonly arise in the pineal region, sellar region, and basal ganglia. Of these tumors, suprasellar ones are more common in females, whereas basal ganglia involvement is seen almost exclusively in males. ICGCTs can be classified into germinomas and non-germinomatous germ cell tumors (NGGCTs) (2, 3). Children may present with a range of symptoms: headache, polydipsia combined with polyuria, decreased sharpness of vision, poor appetite and endocrine problems like precocious puberty or growth delay.

Langerhans cell histiocytosis (LCH) is the most prevalent histiocytic disorder in children, arises from the monocyte-macrophage lineage and can affect any organ system, predominantly bone, skin, and lungs (4, 5). Neurological involvement neurologically presents as one or several infiltrating masses. The hypothalamic-pituitary-adrenal (HPA) axis is one of the most commonly infiltrated sites (6), and the affected children often present to the clinic with poor appetite, dwarfism, polydipsia and polyuria. Therefore, in children with LCH involving the sellar region, a range of clinical manifestations can closely resemble those of germ cell tumors (GCT).

Due to the clinical similarities, MRI provides a more intuitive and economical reference for differentiating between GCT and LCH. In conventional MRI findings, sellar germ cell tumors often present pituitary stalk thickening, absence of the usual T1-weighted high signal in the posterior pituitary, and a sellar-region mass (7). For LCH children with sellar involvement, roughly 70% show stalk thickening, and over time a mass can be developed (8, 9). Therefore, when both diseases are limited to the sellar region, their imaging findings overlap heavily, complicating radiological differentiation. It makes non-invasive preoperative differentiation very challenging. NGGCT can be diagnosed by elevated tumor markers [serum or cerebrospinal fluid AFP ≥25 IU/L and/or *β*-human chorionic gonadotropin (*β*-HCG) ≥50 ng/ml] (2, 10). In contrast, children with negative tumor markers can only be definitively diagnosed through biopsy or diagnostic chemoradiotherapy (11, 12). The diagnostic of LCH is based on the typical clinical symptoms, on clinical imaging and on biopsy of the pathologically altered tissues. Pathological biopsy is the definitive diagnostic method of LCH, typically reveals an infiltrate made up of Langerhans cells, macrophages, lymphocytes, eosinophilic granulocytes, and giant cells (13). However, in clinical practice, because of the characteristics of tumors and their proximity to important neurovascular structures, invasive diagnostic measures are prone to cause serious complications (14). Furthermore, some lesions may yield false-negative results in the early stages of pathological examination, which can affect subsequent diagnosis and treatment. Additionally, the treatment strategies and prognoses differ substantially. Misdiagnosis can lead to inappropriate therapy, disease progression, and irreversible endocrine dysfunction. GCTs typically require platinum-based chemotherapy combined with radiation treatment, and they are highly sensitive to both radiation and chemotherapy (15). For children with germinoma, radiotherapy alone can achieve a 5-year survival rate as high as 90% (16). A multicenter international collaborative study also demonstrated that 71 patients with intracranial germ cell tumors achieved complete remission following chemotherapy alone (17). For the treatment of LCH, given its wide range of sites of origin, the choice between observation, surgical resection, or systemic chemotherapy depends on the extent of the disease. Thus, accurately distinguishing between these two diseases before clinical intervention is critically important, both for developing personalized treatment strategies and for improving patients' long-term outcomes.

Since the concept of radiomics was introduced by Philippe Lambin in 2012 (18), radiomic feature analysis has been widely applied to space-occupying lesions across various systems, offering an in-depth, objective evaluation of tumor phenotypes through tumor heterogeneity analysis (19–21). Radiomics complements conventional imaging by extracting quantitative, sub-visual features that reflect underlying pathophysiology (19). This approach offers precise guidance for tumor differential diagnosis, treatment, and prognosis prediction (22, 23). Its main steps include acquiring imaging data, delineating regions of interest, extracting and filtering features, and building and evaluating models. Moreover, unlike conventional radiology reports, radiomics can add non-semantic image features to the analysis. Studies have shown that biomedical images contain information reflecting underlying pathophysiology (24), and that such relationships can be uncovered through quantitative image analysis. Over the past few years, radiomics has seen growing use in pediatric brain tumor research, for example, to classify germ cell tumors (25), to predict molecular subtypes and prognosis in medulloblastoma (26), and to grade gliomas.

In general, accurate differentiation between sellar GCT and LCH is a cornerstone of effective clinical care. Radiomics offers a way to identify subtle imaging features and distinguish these disorders. It supports clinicians in making accurate diagnoses, avoiding mistakes, and improving outcomes. In this study, we proposed that sellar GCT and LCH differ in their image texture features, and that radiomics analysis can reveal those differences. Consequently, this study aimed to construct MRI-based radiomics models by fusing radiomics features with conventional imaging semantics features as well as clinical information to discriminate LCH from GCT in sellar lesions with negative tumor markers and offer a useful auxiliary diagnosis method.

## 2 Materials and methods

### 2.1 Adherence to checklists

This study adhered to the CLEAR checklist (27). for the reporting of our research. The completed CLEAR checklist has been listed in Supplementary Table S1. The raw data and analysis code that support the findings of this current study are available from the corresponding author on reasonable requests.

### 2.2 Participants and image acquisition

A total of 93 children with germ cell tumors (*n* = 53) and Langerhans cell histiocytosis (*n* = 40) located in the sellar region were retrospectively enrolled in this study between November 2012 and April 2024 at our institution. Patients were stratified into a training set and a test set in an approximate 7:3 ratio, consisting of 65 and 28 patients, respectively. Approval for this protocol was obtained from the Institutional Review Board of our institution. All data used in this study have not been published or used in any other previous publications.

The inclusion criteria of patients were as follows: (1) Lesions involving the sellar and suprasellar regions, with histopathological confirmation of LCH or GCT; (2) Diagnosis of sellar germ cell tumors confirmed by diagnostic therapy; (3) Serum or CSF AFP <25 IU/L and *β*-HCG <50 ng/ml at initial diagnosis. The exclusive criteria were as follows: (1) Absence of preoperative or pre-treatment MRI examination; (2) Images with noise, blurring, or motion artifacts.

All patients underwent pituitary MRI (1.5T or 3.0T scanners) on three different scanners from two manufacturers. There are 35 patients scanned on a 1.5T system (Signa EXCITE HD, GE Healthcare, Chicago, IL, USA), 26 patients scanned on a 3.0T system (Discovery MR750, GE Healthcare, Milwaukee, WI, USA), and 32 patients scanned on another 3.0T system (Achieva, Philips Healthcare, Best, The Netherlands). All examinations were performed using an eight-channel phased-array head coil. Standard sequences included: T1-weighted Imaging (T1WI): Repetition Time (TR) 450–550 ms (1.5T)/550–700 ms (3.0T), Echo Time (TE) 8–20 ms, slice thickness 2–3 mm, FOV160*160 mm; T2-weighted Imaging (T2WI): TR 2,500–4,000 ms, TE 80–120 ms, slice thickness 2–3 mm, FOV160*160 mm. Finally, T1WI and T2WI scans from the pituitary MRI series were obtained for all enrolled participants.

### 2.3 Radiomics workflow

**Figure 1:**
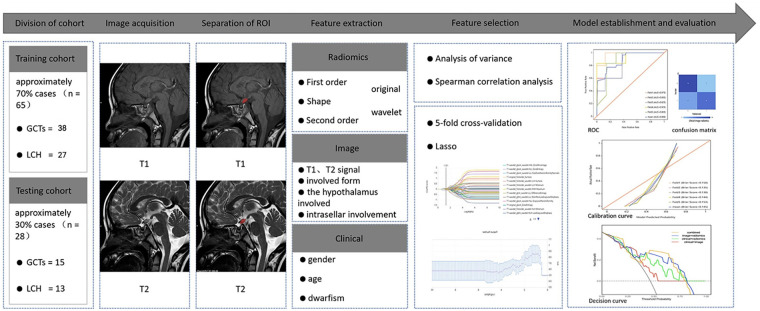
The flowchart of the process of radiomics. It encompasses the stages of image acquisition, feature extraction, feature selection, and model analysis.

The study workflow (Figure 1) included: (1) cohort division into training (*n* = 65, GCT = 38, LCH = 27) and testing (*n* = 28, GCT = 15, LCH = 13) sets; (2) image acquisition using T1WI and T2WI sequences; (3) manual region-of-interests (ROIs) segmentation separately on T1WI and T2WI; (4) extraction of radiomics, imaging semantic, and clinical features; (5) feature selection was conducted through ANOVA, Spearman correlation analysis, 5-fold cross-validation, LASSO regression, and calibration curve assessment; (6) model establishment and evaluation using receiver operating characteristic (ROC) curves, calibration curves, and decision curve analysis (DCA).

### 2.4 Image preprocessing

Image intensity variations were normalized using mean-variance normalization. Gray-level discretization was applied using a fixed bin width of 5. Finally, all images were resampled to an isotropic resolution of 1 × 1 × 1 mm³ using B-spline interpolation for the intensity images and nearest-neighbor interpolation for the segmentation masks. These preprocessing steps were performed before tumors segmentation. No missing data were present in the dataset.

### 2.5 Tumors segmentation and features extraction

ROIs of tumors were manually segmented on all T1WI and T2WI slices by two independent neuroradiologists (≥5 years’ experience), blinded to clinical data and diagnosis, using the uAI (https://urp.united-imaging.com/) (28) Research Portal platform. The ROI included all components of the lesion, namely: the thickened stalk segment, mass or nodule in the infundibulohypothalamic region on consecutive slices, while carefully excluding adjacent normal structures. The segmentations were reviewed and adjudicated by a senior neuroradiologist (15 years' experience).

### 2.6 Radiomic features extraction

The images were processed using both Original and wavelet filters for transformation, and features including first-order, morphological, texture, and transformation-based characteristics were subsequently extracted. Features demonstrating good inter-observer reproducibility (Intraclass Correlation Coefficient, ICC ≥0.8) within the training set were retained. Retained features underwent Z-score normalization. Z-score normalization was performed using the mean and standard deviation calculated from the training set only. These parameters were then applied to both the training and testing sets to ensure consistent scaling while preventing data leakage. All feature selection steps (ANOVA, Spearman correlation, and LASSO with five-fold cross validation) were conducted exclusively on the normalized training set. The testing set was normalized using the training set parameters and used only for final model evaluation, thereby preventing data leakage.

### 2.7 Clinical and imaging semantic features

The Mann–Whitney *U*-test and the chi-square test were used to evaluate the significance of each factor between the two groups. All analyses were performed using RStudio (https://www.R-project.org/) (29).

### 2.8 Model construction

After feature selection using LASSO, hyperparameter tuning was performed using grid search (GridSearchCV) with five-fold stratified cross-validation on the training set. We used three classification models, including Logistic Regression (LR), Random Forest (RF), and Support Vector Machine (SVM), based on imaging semantic features, radiomics features, and clinical features. To handle class imbalance, the class_weight = balanced parameter was used for the SVM and LR classifiers, and class_weight = balanced_subsample was used for the RF classifier. The optimal hyperparameters for each classifier were chosen as the combination that yielded the highest mean cross validation AUC on the training set. After tuning, the three classifiers were compared based on their cross-validation performance, and the best performing classifier was selected for subsequent analysis. Furthermore, integrated models were developed by combining different feature sets, including: imaging semantic with clinical features, radiomic with clinical features, radiomic with imaging semantic features, and all three feature categories together. Finally, 7 models in total were trained on the full training set using these optimal hyperparameters and evaluated on the independent testing set.

Model performance was evaluated on the independent testing set. Receiver Operating Characteristic (ROC) curves were generated, and the Area Under the Curve (AUC) was calculated. Additional metrics included Accuracy (ACC), Sensitivity, Specificity, Precision, F1 score and Youden's Index. They were calculated by using RStudio and the uAI Research Portal. Decision Curve Analysis (DCA) assessed clinical utility. Statistical significance of differences in AUC between models was evaluated using Delong's test. *P*-values were adjusted for multiple comparisons using the False Discovery Rate (FDR).

## 3 Results

### 3.1 Radiomics feature selection

A total of 1,629 radiomics features were initially extracted per patient from T1WI and T2WI sequences. Following assessment of inter-observer reproducibility, 1,020 features (ICC ≥0.8) were retained. Initial screening using ANOVA and Spearman correlation (filtering highly correlated features) reduced the set to 48 features (21 from T1WI, 27 from T2WI). Final selection via LASSO regression with five-fold cross-validation identified 7 robust, stable features (6 from T1WI, 1 from T2WI; voting count ≥3) for model building. The name of radiomics features and filter parameters are showed in Supplementary Table S2.

### 3.2 Significant clinical and imaging semantic features

The demographic and clinical characteristics of the enrolled patients are detailed in Table 1, while the imaging semantic features are summarized in Table 2. Among the clinical variables, age, sex, and dwarfism differed significantly between groups. With respect to imaging semantics, significant differences emerged in T1WI signal intensity, pituitary fossa involvement, hypothalamus involvement, and lesion morphology.

**Table 1:** Demographic and clinical.

Characteristics		Training set			Testing set		
		LCH (*n* = 27)	GCTs (*n* = 38)	*P*-value (<0.05)	LCH (*n* = 13)	GCTs (*n* = 15)	*P*-value (<0.05)
Age		7.04 ± 2.93	9.97 ± 2.86	<0.01	7.74 ± 2.59	9.31 ± 2.53	0.11
Gender	Male	20	16	0.01	6	7	0.978
	Female	7	22		7	8	
Polyuria polydipsia		19	34	0.139	9	13	1
Dwarfism		7	0	0.035	1	6	0.179
Headache		2	7	0.287	1	0	0.40
Poor appetite		3	8	0.498	0	6	0.051

**Table 2:** Image semantic features.

Characteristics			Training set			Testing set		
			LCH (*n* = 27)	GCTs (*n* = 38)	*P*-value (<0.05)	LCH (*n* = 13)	GCTs (*n* = 15)	*P*-value (<0.05)
T1WI	Hypo		0	8	<0.01	0	2	0.60
	Iso		23	30		12	13	
	Hyper		4	0		1	0	
T2WI	Hypo		8	4	0.09	4	1	0.21
	Iso		17	27		9	11	
	Hyper		2	7		0	3	
Pituitary fossa involvement			2	12	0.03	0	9	<0.01
Hypothalamus involvement			3	17	<0.01	3	5	0.686
Lesion Morphology	uniform		5	11	0.01	11	5	0.09
	non-uniformity		19	12		0	5	
	space- occupying	1–2 cm	2	8		1	2	
		>2 cm	1	7		1	3	

### 3.3 Model evaluation

**Figure 2:**
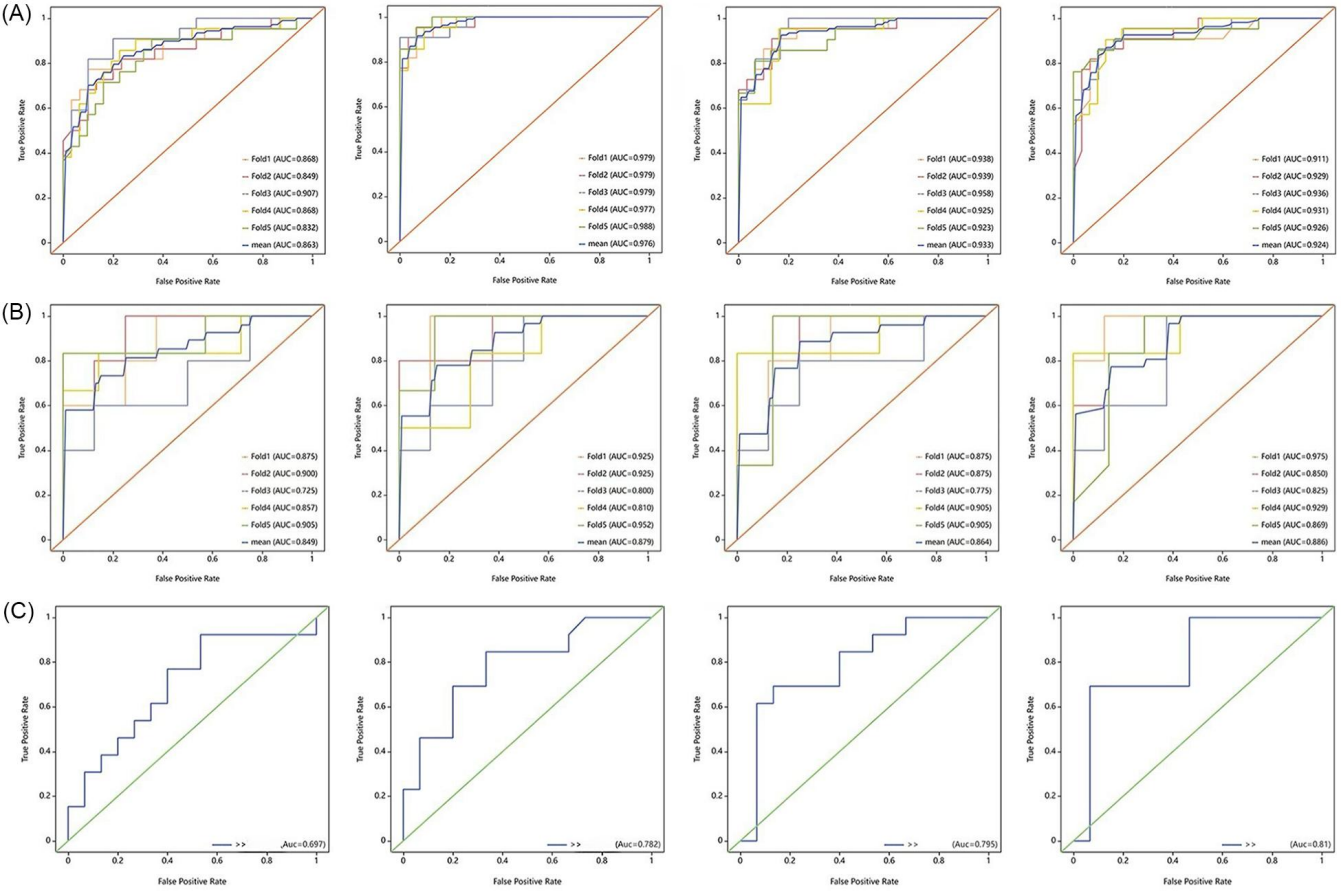
The ROC of the four combined models. **(A)** Performance of ROC curves for clinical + imaging semantic model, clinical + radiomics model, imaging semantic + radiomics model, and clinical + imaging semantic + radiomics model in the training set. **(B)** Performance of ROC curves for clinical + imaging semantic model, clinical + radiomics model, imaging semantic + radiomics model, and clinical + imaging semantic + radiomics model in the validation set. **(C)** Performance of ROC curves for clinical + imaging semantic model, clinical + radiomics model, imaging semantic + radiomics model, and clinical + imaging semantic + radiomics model in the testing set.

**Figure 3:**
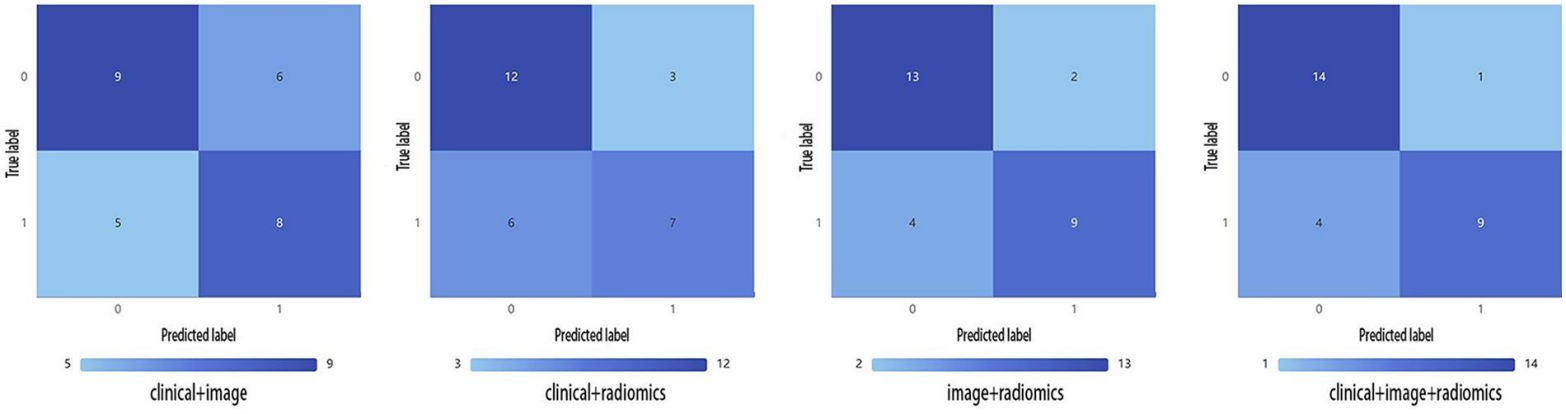
Confusion matrix of the four combined models.

**Figure 4:**
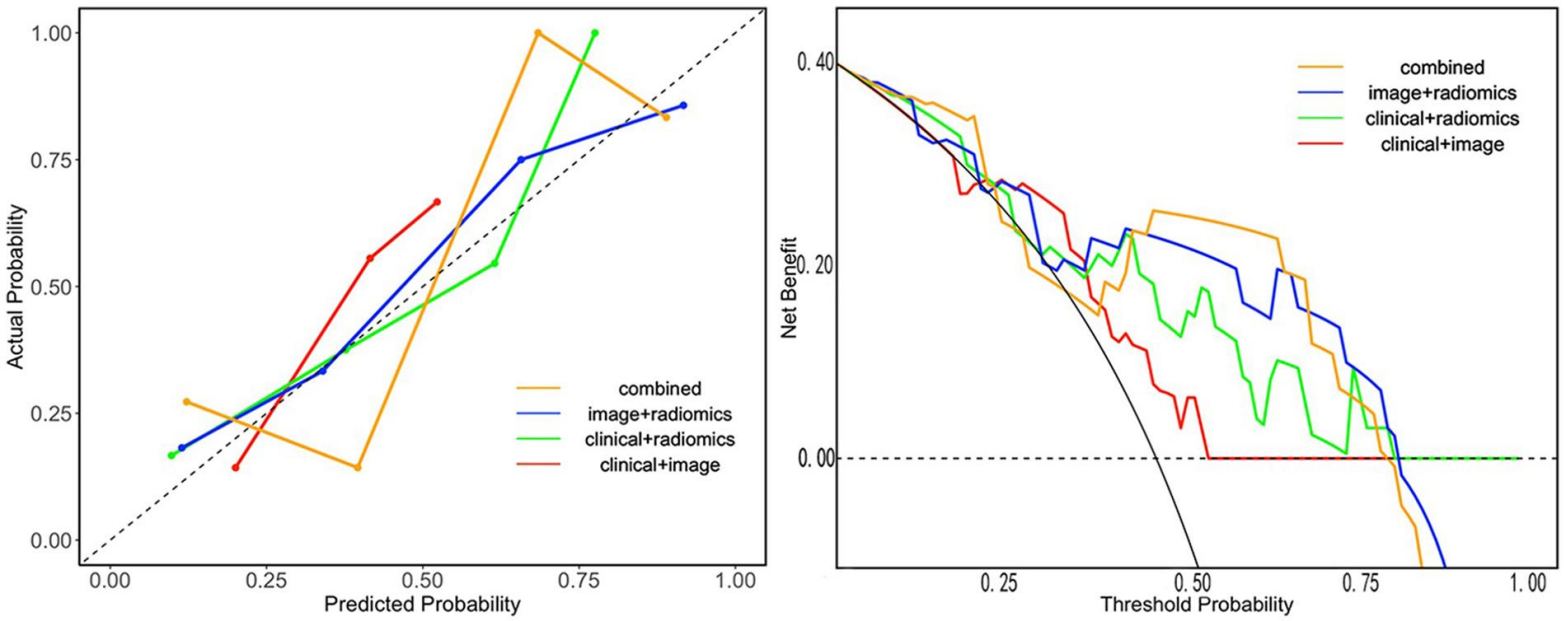
The decision curves and calibration curves of the four combined models.

The search spaces for each classifier are detailed in Supplementary Table S3. The optimized parameters of the model, based on five-fold cross-validation were used for testing the test set. We developed seven models under single and combined conditions, and tested them using the testing set. For each model, we calculated the AUC value and recorded the ACC, Sensitivity, Specificity, Precision, F1 score and Youden's Index. The best-performing classifier for each model in the training set, along with its corresponding performance in the test set, is shown in Table 3. The diagnostic performance of the combined model of radiomics, imaging semantics, and clinical features using the RF classifier was the best, achieving an AUC value of 0.81 in the test set. Additionally, we visually compared the test performance of the four combined models through their ROC curves, confusion matrix, decision curves and calibration curves, as presented in Figures 2–4. Delong's test indicated that the performance of the combine model—radiomic with imaging semantic features and clinical features was significantly superior to that of other combined models (Supplementary Table S4).

**Table 3:** Model performance evaluation.

Model	Method	Training AUC	Validation AUC	Testing					
				AUC	ACC	F1 score	sensitive	specificity	YI
Clinical	LR	0.893	0.865	0.651	0.571	0.538	0.538	0.6	0.138
Image	LR	0.851	0.817	0.705	0.714	0.714	0.769	0.667	0.436
Radiomics	SVM	0.937	0.907	0.821	0.786	0.727	0.615	0.933	0.548
Clinical + image	LR	0.863	0.849	0.697	0.607	0.593	0.615	0.6	0.215
Clinical + radiomics	RF	0.976	0.879	0.782	0.679	0.609	0.538	0.8	0.338
Image + radiomics	LR	0.933	0.864	0.795	0.786	0.75	0.692	0.867	0.559
Clinical + image + radiomics	RF	0.924	0.886	0.81	0.821	0.783	0.692	0.933	0.625

## 4 Discussion

To our knowledge, this study is the first to classify pediatric GCT and LCH using a radiomics approach. In our study, we employed various machine learning methods to establish classification models based on radiomic features, semantic imaging features, and clinical characteristics, to differentiate between GCT and LCH. Our results demonstrate that the combined model integrating radiomics, imaging semantic features, and clinical variables achieved the best diagnostic performance, with an AUC of 0.81 in the independent test set. This multimodal approach highlights the potential of radiomics as a non-invasive adjunctive tool for differential diagnosis in clinically challenging scenarios.

Several previous studies have applied radiomics to assess pediatric brain tumors, but few have focused on distinguishing pituitary germ cell tumors from LCH. Chen et al. (30) demonstrated that radiomics-based machine learning aids in the preoperative differentiation between GCTs and craniopharyngiomas. They extracted 40 radiomic features from contrast-enhanced T1-weighted imaging (CE-T1WI) and T2WI, and built nine models using three classification algorithms: Linear Discriminant Analysis (LDA), Support Vector Machine (SVM), Random Forest (RF). The best predictive model was built using RF with LASSO based on the CE-T1WI sequence, with an AUC of 0.91. Fan et al.'s (31) study aimed to differentiate pineal germinomas from pineoblastomas by combining radiomics with clinical features, using a clinical imaging model. They retrospectively analyzed 134 patients with pineal region tumors, including 69 germinomas and 65 pineoblastomas. Radiomic features were extracted from MR images, and seven features were selected to construct a radiomic model. This model achieved AUC values of 0.920 and 0.880 in the training and validation sets, respectively. The final fusion model including radiomics combined with four selected clinical features showed great discrimination and calibration ability, with AUC values for the training set and validation set being 0.950 and 0.940, respectively. The model's good performance and high sensitivity in distinguishing between germ cell tumor and pinealoblastoma was demonstrated by this study, which may be helpful in developing an individualized treatment regimen for patients with pineal region tumors.

In our study, across all models, the clinical + radiomics model with RF achieved the highest training AUC (0.976), yet its performance dropped substantially in the testing set (AUC = 0.782), indicating marked overfitting. This is likely attributable to the high complexity of the Random Forest classifier combined with the relatively modest sample size. Conversely, the radiomics-only model with SVM obtained the highest testing AUC (0.821) but exhibited lower sensitivity (0.615) and a lower F1-score (0.727) compared to the combined model (clinical + image + radiomics). Although its testing AUC was marginally higher than that of the combined model (0.81), the combined model was ultimately selected as the best overall model based on several clinically relevant considerations. First, the combined model achieved a better balance between sensitivity (0.692) and specificity (0.933) compared to the radiomics-only model (sensitivity 0.615, specificity 0.933), offering higher sensitivity to avoid missed diagnoses. Second, the combined model demonstrated a higher F1-score (0.783 vs. 0.727) and higher accuracy (0.821 vs. 0.786), reflecting more reliable overall classification. Third, it demonstrated superior calibration and a more favorable net benefit on decision curve analysis, reflecting greater clinical utility. At last, the integration of readily available clinical and imaging semantic features enhances interpretability and clinician trust, addressing the “black-box” concern often associated with pure radiomics signatures. Thus, while the radiomics-only model achieved a slightly higher testing AUC, the combined model was preferred for its balanced performance, better calibration, and practical applicability. This highlights the importance of evaluating models using multiple metrics—including calibration, clinical utility, and generalizability—rather than relying solely on AUC.

In the radiomic features, we found that after feature selection, the retained features predominantly belonged to transformed features, with texture features being the majority. These included GLCM, GLRLM, and GLSZM. Texture features reflect the degree of gray intensity fluctuation or sudden change in the image (23, 32). Among these, GLCM provides descriptions of entropy, gray level nonuniformity, and other related information, while GLRLM and GLSZM quantify the number of adjacent pixels or voxels with the same gray level (33). These texture features reflect differences in morphology, structure, and texture within different tissues. For example, Skogen et al. found that coarse texture entropy and uniformity could differentiate between low-grade and high-grade tumors (34). Applying these interpretations to our cohort, the higher textural complexity observed in GCTs may be attributable to their known histopathological characteristics. GCTs often contain mixed germ cell components, including germinoma with syncytiotrophoblastic cells, teratomatous elements, or yolk sac tumor components, which contribute to greater cellular pleomorphism and architectural disarray. Conversely, LCH lesions are often characterized by a more uniform infiltration of Langerhans cells, which might result in smoother texture. These histopathological differences may underlie the distinct radiomic texture signatures captured by our model.

In recent years, radiomic feature analysis has been widely applied to lesions in various systems, providing an in-depth, objective assessment of tumor phenotypes through the heterogeneous characteristics of tumors (24, 35–37). This method offers precise guidance for tumor differential diagnosis, treatment, and prognosis prediction. Our study confirms that radiomics-based differentiation between these two diseases is feasible. Additionally, due to the relatively low incidence rate, we collected imaging, clinical, and pathological data from 53 cases of sellar GCTs and 40 cases of LCH at a single center for this radiomics study. This collection of data from a single center is invaluable, and the large sample size in this study provides more reliable results compared to previous scattered case studies. Finally, the final fusion model based on clinical characteristics combined with radiomics showed better performance and generalization ability.

The study is also limited in several aspects. It is the first-time single-center, retrospective study without external validation, the generalizability of our model remains uncertain. Center-specific factors, including patient selection, imaging protocols and segmentation practices, may affect the results. Second, the rarity of the diseases limited the sample size. Without a formal sample size calculation, the cohort was defined by case availability over 12 years. The smaller class (LCH, *n* = 40) yielded an EPV of 5.7, below the recommended minimum of 10 for a 7-feature model, which may explain the observed overfitting. Nevertheless, the testing AUC of 0.81 indicates acceptable discriminative performance. We recognize these as the limitations of the current work. Larger multi-center studies are needed to confirm these findings and to support clinical translation.

## 5 Conclusion

Radiomics feature analysis can serve as an important adjunct to the differential diagnosis of sellar GCTs and LCH in the absence of tumor markers. In conclusion, the integrated model based on radiomics and semantic image and clinical characteristics has good performance and high sensitivity in differential diagnosis of the above two diseases, which is helpful for the development of an individualized treatment plan for patients.

## Data Availability

The original contributions presented in the study are included in the article/Supplementary Material, further inquiries can be directed to the corresponding author/s.
