# Supplementary material for: Machine learning based on radiomics for discriminating sellar region langerhans cell histiocytosis from germ cell tumors
Source: Front Pediatr. 2026 Apr 24;14:1775150. doi: 10.3389/fped.2026.1775150 (PMC13153032; doi:10.3389/fped.2026.1775150)
Supplement: Supplementary file 1 [file Table1.docx]

Supplementary Material

# Supplementary Tables

## Supplementary. Table 1. CheckList for EvaluAtion of Radiomics research (CLEAR checklist)

| **Section** | **No** | **Item** | **Yes** | **No** | **n/a** | **Page** |  |
| --- | --- | --- | --- | --- | --- | --- | --- |
| **Title** | | | | | | | |
|  | 1 | Relevant title, specifying the radiomic methodology | ☑ | ☐ | ☐ | 1 |  |
| **Abstract** | | | | | | | |
|  | 2 | Structured summary with relevant information | ☑ | ☐ | ☐ | 1 |  |
| **Keywords** | | | | | | | |
|  | 3 | Relevant keywords for radiomics | ☑ | ☐ | ☐ | 1 |  |
| **Introduction** | | | | | | | |
|  | 4 | Scientific or clinical background | ☑ | ☐ | ☐ | 2 |  |
|  | 5 | Rationale for using a radiomic approach | ☑ | ☐ | ☐ | 2 |  |
|  | 6 | Study objective(s) | ☑ | ☐ | ☐ | 2 |  |
| **Method** | | | | | | | |
| *Study Design* | 7 | Adherence to guidelines or checklists (e.g., CLEAR checklist) | ☑ | ☐ | ☐ | 4 |  |
|  | 8 | Ethical details (e.g., approval, consent, data protection) | ☑ | ☐ | ☐ | 4 |  |
|  | 9 | Sample size calculation | ☐ | ☐ | ☑ |  |  |
|  | 10 | Study nature (e.g., retrospective, prospective) | ☑ | ☐ | ☐ | 4 |  |
|  | 11 | Eligibility criteria | ☑ | ☐ | ☐ | 4 |  |
|  | 12 | Flowchart for technical pipeline | ☑ | ☐ | ☐ | 4 |  |
| *Data* | 13 | Data source (e.g., private, public) | ☑ | ☐ | ☐ | 4 |  |
|  | 14 | Data overlap | ☑ | ☐ | ☐ | 4 |  |
|  | 15 | Data split methodology | ☑ | ☐ | ☐ | 4 |  |
|  | 16 | Imaging protocol (i.e., image acquisition and processing) | ☑ | ☐ | ☐ | 4 |  |
|  | 17 | Definition of non‑radiomic predictor variables | ☑ | ☐ | ☐ | 4 |  |
|  | 18 | Definition of the reference standard (i.e., outcome variable) | ☑ | ☐ | ☐ | 4 |  |
| *Segmentation* | 19 | Segmentation strategy | ☑ | ☐ | ☐ | 5 |  |
|  | 20 | Details of operators performing segmentation | ☑ | ☐ | ☐ | 5 |  |
| *Pre-processing* | 21 | Image pre‑processing details | ☑ | ☐ | ☐ | 4,5 |  |
|  | 22 | Resampling method and its parameters | ☑ | ☐ | ☐ | 4 |  |
|  | 23 | Discretization method and its parameters | ☑ | ☐ | ☐ | 4 |  |
|  | 24 | Image types (e.g., original, filtered, transformed) | ☑ | ☐ | ☐ | 5 |  |
| *Feature extraction* | 25 | Feature extraction method | ☑ | ☐ | ☐ | 5 |  |
|  | 26 | Feature classes | ☑ | ☐ | ☐ | 6 |  |
|  | 27 | Number of features | ☑ | ☐ | ☐ | 6 |  |
|  | 28 | Default configuration statement for remaining parameters | ☐ | ☐ | ☑ |  |  |
| *Data preparation* | 29 | Handling of missing data | ☐ | ☐ | ☑ |  |  |
|  | 30 | Details of class imbalance | ☑ | ☐ | ☐ | 5 |  |
|  | 31 | Details of segmentation reliability analysis | ☑ | ☐ | ☐ | 5 |  |
|  | 32 | Feature scaling details (e.g., normalization, standardization) | ☑ | ☐ | ☐ | 5 |  |
|  | 33 | Dimension reduction details | ☑ | ☐ | ☐ | 5 |  |
| *Modeling* | 34 | Algorithm details | ☑ | ☐ | ☐ | 5 |  |
|  | 35 | Training and tuning details | ☑ | ☐ | ☐ | 5 |  |
|  | 36 | Handling of confounders | ☑ | ☐ | ☐ | 5 |  |
|  | 37 | Model selection strategy | ☑ | ☐ | ☐ | 5 |  |
| *Evaluation* | 38 | Testing technique (e.g., internal, external) | ☑ | ☐ | ☐ | 5 |  |
|  | 39 | Performance metrics and rationale for choosing | ☑ | ☐ | ☐ | 6 |  |
|  | 40 | Uncertainty evaluation and measures (e.g., confidence intervals) | ☐ | ☑ | ☐ |  |  |
|  | 41 | Statistical performance comparison (e.g., DeLong’s test) | ☑ | ☐ | ☐ | 6 |  |
|  | 42 | Comparison with non‑radiomic and combined methods | ☑ | ☐ | ☐ | 6 |  |
|  | 43 | Interpretability and explainability methods | ☐ | ☑ | ☐ |  |  |
| **Results** | | | | | | | |
|  | 44 | Baseline demographic and clinical characteristics | ☑ | ☐ | ☐ | 6 |  |
|  | 45 | Flowchart for eligibility criteria | ☐ | ☑ | ☐ |  |  |
|  | 46 | Feature statistics (e.g., reproducibility, feature selection) | ☑ | ☐ | ☐ | 6 |  |
|  | 47 | Model performance evaluation | ☑ | ☐ | ☐ | 6 |  |
|  | 48 | Comparison with non‑radiomic and combined approaches | ☑ | ☐ | ☐ | 6 |  |
| **Discussion** | | | | | | | |
|  | 49 | Overview of important findings | ☑ | ☐ | ☐ | 6,7 |  |
|  | 50 | Previous works with differences from the current study | ☑ | ☐ | ☐ | 7,8 |  |
|  | 51 | Practical implications | ☑ | ☐ | ☐ | 7,8 |  |
|  | 52 | Strengths and limitations (e.g., bias and generalizability issues) | ☑ | ☐ | ☐ | 8 |  |
| **Open Science** | | | | | | | |
| *Data availability* | 53 | Sharing images along with segmentation data [n/e] | ☑ | ☐ | ☐ | 9 |  |
|  | 54 | Sharing radiomic feature data | ☑ | ☐ | ☐ | 9 |  |
| *Code availability* | 55 | Sharing pre‑processing scripts or settings | ☑ | ☐ | ☐ | 4 |  |
|  | 56 | Sharing source code for modeling | ☑ | ☐ | ☐ | 4 |  |
| *Model availability* | 57 | Sharing final model files | ☑ | ☐ | ☐ | 9 |  |
|  | 58 | Sharing a ready‑to‑use system [n/e] | ☐ | ☐ | ☑ |  |  |

**Yes**, details provided; **No**, details not provided; **n/e**, not essential; **n/a**, not applicable

## Supplementary. Table 2. The name of radiomics features and filter parameters

| Radiomic Features | | Filters Parameters |
| --- | --- | --- |
|  |  | Wavelet |
| Shape | 2D=10、3D=16 | LLH、LHL  LHH、HLL、HLH  HHL、HHH、LLL |
| Firstorder | 19 |  |
| Second-order  features | gray-levelco-occurrencematrix（GLCM）=24  gray-levelrun-lengthmatrix（GLRLM）=16  gray-levelsize-regionmatrix（GLSZM）=16  adjacentgray-leveldifferencematrix（NGTDM）=5  gray-leveldependencematrix（GLDM）=14 |  |

## Supplementary. Table 3. The optimised parameters of each classifier

| Methods | Parameters | radiomics | clinical | image | radiomics + clinical | Image + radiomics | clinical +image | clinical + image + radiomics |
| --- | --- | --- | --- | --- | --- | --- | --- | --- |
| SVM | C | 2.0 | 10.0 | 1.0 | 9.0 | 5.0 | 1.0 | 9.0 |
|  | CW | balanced | balanced | balanced | balanced | balanced | balanced | balanced |
|  | Ga | 0.01 | 0.05 | 0.05 | 0.01 | 0.1 | 0.05 | 0.01 |
|  | Ke | poly | poly | sigmoid | sigmoid | sigmoid | rbf | sigmoid |
| RF | CW | balanced_subsample | balanced_subsample | balanced | balanced | balanced | balanced | balanced_subsample |
|  | Cr | gini | entropy | entropy | gini | gini | gini | entropy |
|  | MD | 256 | 7 | 64 | 32 | 8 | 5 | 28 |
|  | MSL | 4 | 9 | 2 | 2 | 4 | 12 | 12 |
|  | MSS | 2 | 4 | 9 | 10 | 12 | 11 | 15 |
|  | NE | 5 | 10 | 10 | 20 | 10 | 20 | 20 |
| LR | C | 4.0 | 0.05 | 1.5 | 4.0 | 1.0 | 0.05 | 0.5 |
|  | CW | balanced | balanced | balanced | balanced | balanced | balanced | balanced |
|  | Pe | l2 | l2 | l2 | l2 | l2 | l2 | l2 |
|  | Tol | 1e-05 | 1e-05 | 1e-05 | 1e-05 | 1e-05 | 1e-05 | 1e-05 |

## Supplementary. Table 4. Delong test between models

| Models | DeLong | FDR |
| --- | --- | --- |
| clinical + image + radiomics VS clinical + image | 0.009 | 0.027 |
| clinical + image + radiomics VS clinical + radiomics | 0.489 | 0.702 |
| clinical + image + radiomics VS image + radiomics | 0.702 | 0.702 |

**
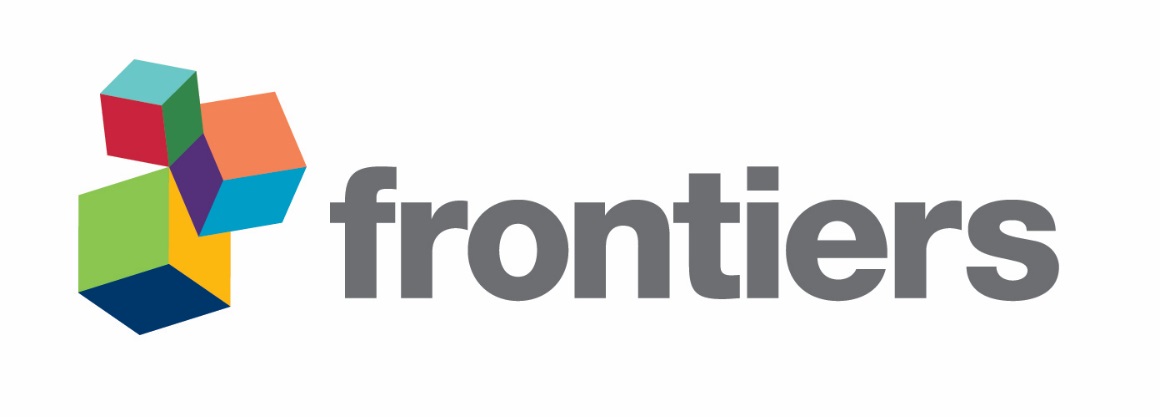
**
